# Effects of *Echinacea purpurea* on Hepatic and Renal Toxicity Induced by Diethylnitrosamine in Rats

**Published:** 2013-05-04

**Authors:** Annahita Rezaie, Ali Fazlara, Mojtaba Haghi Karamolah, Ali Shahriari, Hossein Najaf Zadeh, Marzieh Pashmforosh

**Affiliations:** 1 Department of Pathobiology, Faculty of Veterinary Medicine, Shahid Chamran University of Ahvaz, Ahvaz, IR Iran; 2 Department of food Hygiene, Faculty of Veterinary Medicine, Shahid Chamran University of Ahvaz, Ahvaz, IR Iran; 3 Department of Basic Sciences, Faculty of Veterinary Medicine, Shahid Chamran University of Ahvaz, Ahvaz, IR Iran; 4 School of Pharmacy, Jundishapur University of Medical Sciences, Ahvaz, IR Iran; 5 Research Committee, Jundishapur University of Medical Science, Ahvaz, IR Iran

**Keywords:** Diethylnitrosamine, Echinacea, Rats, Liver, Kidney, Toxicity

## Abstract

**Background:**

Nitrites are mainly used in food preservation. These materials could change to nitrosamine due to the effect of heat and gastric acid. Nitrosamine is absorbed in intestine and enters the liver and hepatocytes by portal venous system, and hampers the detoxification system of liver by interfering in cytochrome P450 enzymes, so, the liver gently proceeds to cirrhosis and cancer.

**Objectives:**

The current study aimed to investigate the hepatic and renal protective effects of aerial parts of *Echinacea purpurea* extract (EPE) on injury induced by diethylnitrosamine (DEN).

**Materials and Methods:**

Twenty Wistar rats were divided into 4 groups. Groups were as follow: Control group (normal saline), DEN (200 mg/kg, IP, a single dose), EPE (100 mg/kg, orally, daily) and DEN + EPE which received as group DEN and EPE. After 30 days, Blood samples, and liver and kidney tissues were taken for further examination. Aspartate transaminase (AST), alanine transaminase (ALT), alkaline phosphatase (ALP), BUN, Creatinine and total and direct bilirubin were estimated in serum.

**Results:**

DEN induced hepatotoxicity and nephrotoxicity in all the treated animals by elevated serum ALT, AST, ALP and BUN, creatinin and total and direct bilirubin levels. AST, BUN and total and direct bilirubin significantly decreased in DEN + EPE compared to DEN group. After 30 days of DEN administration, histopathological investigation revealed proliferation of hepatic stellate cells and early fibrosis which were partly improved by EPE administration.

**Conclusions:**

The current study findings indicated that *Echinacea purpurea* extract played an important role in the protection against DEN toxicity in rats.

## 1. Background

Nitrites are mainly used in food preservation. These materials could change to nitrosamine due to the effect of heat and gastric acid. Nitrosamine is absorbed in intestine and enters the liver and hepatocytes by portal venous system, and hampers the detoxification system of liver by interfering in cytochrome P450 enzymes, so, the liver gently proceeds to cirrhosis and cancer (1). Diethylnitrosamine (DEN), a hepatocarsinogen and hepatotoxin, is synthesized endogenously and found in work place, processed meats, tobacco smoke, soybean, cheese and wide variety of foods also it is produced from metabolism of some drugs (2). It is reported that DEN cause oxidative stress during the metabolism that lead to cytotoxicity, mutagenicity and carcinogenicity (3, 4). DEN is biotransformed by mixed-function cytochrome P450 dependent monooxidase systems and its metabolic activation is responsible for the onset of the toxic effects (5). So the use of antioxidants offer to protect this deleterious effects. *Echinacea purpurea*, one of the most important medical herbs, has been used to treat common cold and infection disease. It contains a variety of medically important substances that play a role in its therapeutic effects which include alkylamides, caffeic acid derivatives, glycoproteins, polysaccharides, polyacetylenes, phenolic compounds, cinnamic acids, essential oils and flavonoids (6, 7). Several phenolic compounds have been reported to be inhibitors of chemical carcinogenesis and mutagenesis (8). *Echinacea purpurea* has many beneficial features, especially activation of immune system by increasing the number of circulating white blood cells, stimulating phagocytosis, T-cell production, lymphocytic activity, cytokine production, cellular respiration, activity against tumor cell, inhibiting hyaluronidase enzyme secretion and trigger the alternate complement pathway (9-12). In previous studies, anti-inflammatory effects of this extract have been investigated in Arsenic induced hepatic toxicity (13).

## 2. Objectives

The current study aimed to investigate the hepatoprotective effects of *Echinacea purpurea* extract on liver damaged induced by DEN.

## 3. Materials and Methods

### 3.1. Materials

Diethylnitrosamine (Sigma Aldrich, USA), and hydro-alcoholic extract of aerial parts of *Echinacea purpurea* (Goldaruo Co. Isfahan, Iran).

### 3.2. Animals

Twenty male Wistar rats, weighting 180-200g were used. Rats were obtained from the central laboratorial animal facility at the faculty of medicine of the Jundishapur University, Ahvaz, Iran. The rats were housed in cages under controlled environmental conditions (25 ºC and a 12 h light/dark cycle) that had free access to standard rat pellet food and tap water. After one week acclimatization, the rats were divided into 4 equal groups (5 rats each). The groups were tested as follows: Control group, without receiving DEN or EPE (negative control), DEN group, a single dose of 200 mg/kg DEN intraperitoneally (14), EPE Group, and 100 mg/kg of EPE orally for 30 days (13) and DEN + EPE Group, a single dose of DEN and also oral EPE with the same dose was prescribed to DEN and EPE groups. At the end of 30 days, rats were exsanguinated through cardiac puncture after a 12h fast (water ad libitum). Sera concentrations of ALT, AST and ALP were assessed as a measure of hepatic cell damage using corresponding commercial kits (Pars Azmoon, Tehran, Iran) according to manufacturer’s instruction. Creatinin (Jaffe method), direct and total bilirubin (DCA method) of sera were measured for evaluating kidney by commercial kits (Pars Azmoon, Tehran, Iran). The livers and kidneys were extracted and fixed for preparation of histopathologic sections.

### 3.3. Histopathological Evaluation

Specimens were processed routinely in 10% formalin buffer, and embedded in paraffin. Tissue sections of 4 µm were obtained, and stained with haematoxylin and eosin (H & E). For further examination of liver section, Masson's trichrome (MT) staining was carried out. Histopathological examinations were performed under a light microscope. All histopathological examinations were performed by a pathologist, who was blinded to all groups of tissue specimens.

### 3.4. Statistical Analysis

The statistical analysis of the data was done by the one way Analysis of Variance (ANOVA) followed by Tukey’s multiple comparison tests using Sigma Stat 2. (Systat Software. Inc Point Richmond. CA). Result values Mean ± SE, with level of significance at *P* ≤ 0.05.

## 4. Results

### 4.1. Biochemical Results

No significant change was observed in ALP and ALT levels between different groups (*P* > 0.05). The serum AST levels were significantly higher in DEN group as compared to those of control, EPE and DEN + EPE groups (*P* ˂ 0.05). Whereas EPE and DEN + EPE treated rats did not show any significant change in serum AST level but the enzyme values decreased in them. The serum BUN level showed significant increase in DEN treated rats as compared with control and EPE treated animals (*P* ˂ 0.05) (Table 1). It was found that creatinine levels significantly decreased in EPE treated rats compared with the DEN , control , and EPE groups almost completely suppressed. DEN induced the increases in creatinine levels. The total bilirubin levels were found to be approximately five folds higher in DEN treated rats compared with control group similarly, the direct bilirubin levels increased significantly in DEN treated rats compared with the control, EPE, DEN + EPE groups (Table 2).

**Table 1.** Biochemical Enzymes in the Sera of Experimental Animals

	Control a a	DEN b a	DEN + EPE c a	EPE d a
**ALT (SGPT), U/L**	75.90 ± 13.06	72.87 ± 6.92	64.27 ± 13.38	46.14 ± 7.60
**AST (SGOT), U/L**	73.72 ± 5.06	196.12 ± 19.06 a, c, d	110.35 ± 28.33	115.39 ± 8.30
**ALP, U/L**	1154.08 ± 25.98	1530.87 ± 199.30	1419.30 ± 74.95	1467.80 ± 123.10

^a^Values Expressed as Mean ± SEM of Animals in Each Group. Different Letters Show Significant Difference Between Groups (P < 0.05)

**Table 2.** Biochemical Parameters in the Sera of Experimental Animals a

	Control a a	DEN b a	DEN ± EPE c a	EPE d a
**BUN, mg/dL**	19.82 ± 0.69	29.06 ± 0.71 a, d	27.22 ± 3.17	21.92 ± 2.09
**Creatinine, mg/dL**	147.93 ± 10.39 d	165.98 ± 22.60 d	123.28 ± 21.42	87.33 ± 10.69
**Total Bilirubin, mg/dL**	11.14 ± 1.78	53.45 ± 8.67 a, c, d	31.55 ± 6.30 a	28.71 ± 2.15
**Direct Bilirubin, mg/dL**	7.54 ± 1.12 d	18.85 ± 1.95 a, c, d	5.21 ± 0.18	1.26 ± 0.10

^a^Values Expressed as Mean ± SEM of Animals in Each Group. Different Letters Show Significant Difference Between Groups (P < 0.05)

### 4.2. Histopathological Results

**Figure 1.**
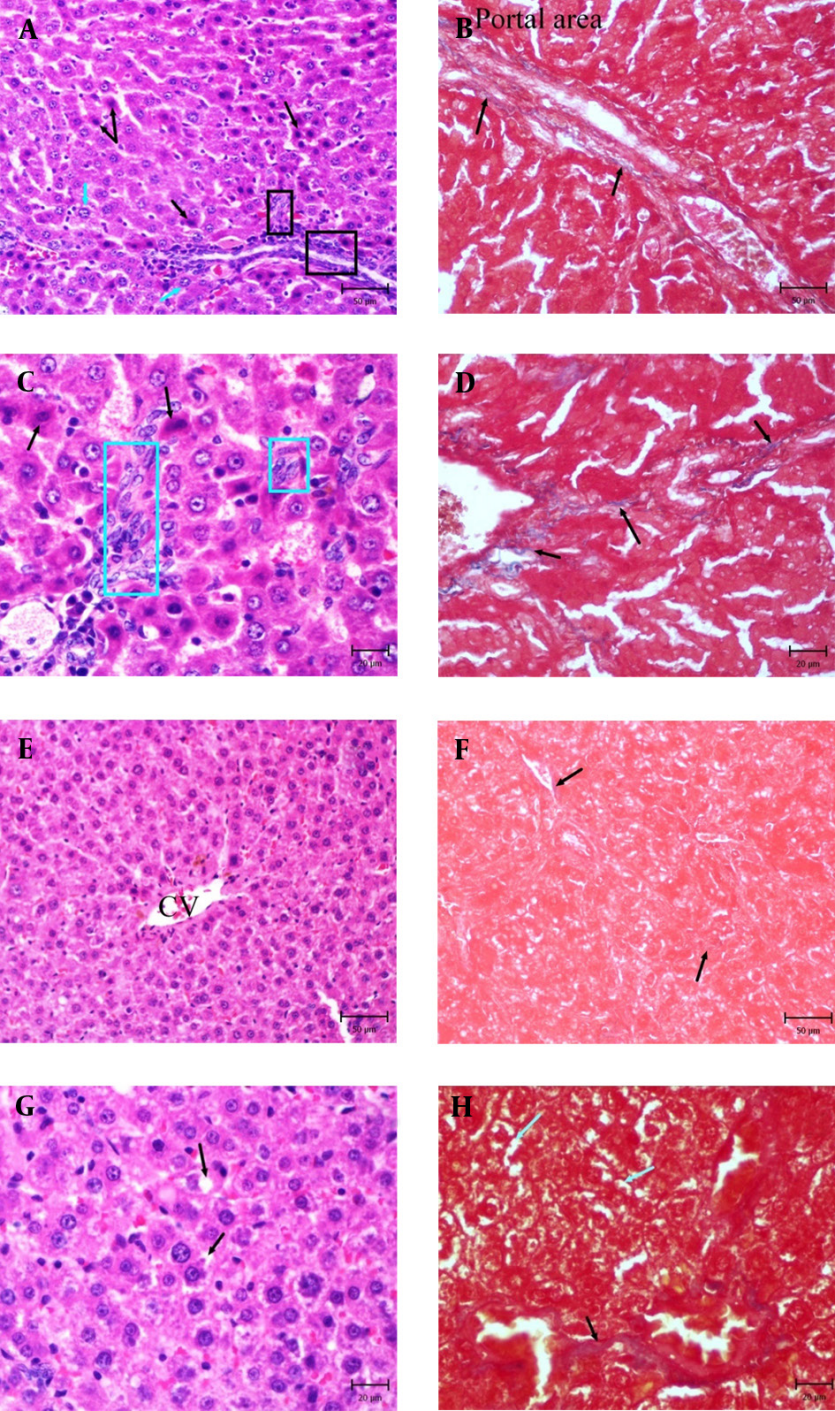
Photomicrographs of Liver in DEN and DEN + EPE Experimental Groups **A.** DEN group, note to proliferation of hepatic stellate cells (Square), necrotic hepatocytes (Black arrows) and hepatocytes with enlarged nuclei (Karyomegali) (Blue arrows) (H & E, Bar: 50µm). **B.** DEN group, note to blue fibers around portal area (MT staining, Bar: 50 µm). **C.** Part of picture A with high magnification, proliferated stellate cells is obvious (Blue square) (H & E, Bar: 20 µm). **D.** Part of picture C, note to increased blue fibers (MT staining, Bar: 50 µm). **E.** DEN + EPE group, note to retained Architecture of liver (H & E, Bar: 50 µm). **F.** DEN + EPE group, normal rate of Blue materials (MT Staining, Bar: 50 µm). **G.** Part of picture E, note to cell swelling (Black arrows) in hepatocytes (H & E, Bar: 20 µm). **H.** Part of picture F, cell swelling in hepatocytes (Blue arrows) and blue fibers (Black arrows) are obvious (MT staining, Bar: 20 µm).

Livers of control and EPE rats showed normal lobular architecture with central vein and radiating cords of hepatocytes. Hepatocytes were polyhedral in shape and their cytoplasm was granulated with small uniform nuclei. DEN group exhibited extensive cell swelling and single cell necrosis. Necrotic cells were small with basophilic nuclei and dark cytoplasm. Dysplastic hepatocytes were observed with enlarged nuclei (karyomegali) and multiple nucleoli in the liver sections of rats treated with DEN. Mild bile duct hyperplasia was evident. There were marked proliferation of hepatic Stellate cells (HSCs) in portal area and also focal proliferation of HSCs was observed (Figure 1A and C). In DEN + EPE rats, the integrity of the hepatocytes was relatively well preserved. The number of necrotic cells, dysplastic hepatocytes and proliferated HSCs reduced which showed a pattern of recovery (Figure 1F and G). The extent of fibrosis was further documented using MT staining in liver sections. In DEN group, accumulation of blue material was seen in portal area and between central vein and portal area (Figure 1B and D). These materials are related to collagen fibers and early fibrosis. In contrast, the density of mentioned material in DEN + EPE group decreased (Figure 1F and H).

Histopathological study of kidney sections revealed acute tubular necrosis in DEN group. There were less necrotic cells in proximal tubules of DEN + EPE. No histopathological changes were observed in control and EPE groups.

## 5. Discussion

DEN is reported to be a well known hepatotoxin and hepatocarcinogen. In the present investigation, DEN induced hepatocellular damage is clearly evidenced by the marked elevation in the activity of serum AST, ALT and ALP which is in agreement with other researches (4, 5, 14-16). These enzymes are the most sensitive markers employed in the diagnosis of hepatic damage because they are cytoplasmic in location and are released into the circulation after cellular damage (3, 17). Current study demonstrated that treatment with EPE for 30 days reduced different parameters in EPE + DEN group and these decreases were significant in AST, BUN and total and direct bilirubin (*P* < 0.05). These findings indicated that EPE partially recovered side effects of DEN. The biochemical findings are supported by histopathological observations of the liver. Microscopic results showed different lesions including necrosis, early hepatic fibrosis with increased synthesis and deposition of connective tissue and dysplastic hepatocytes with large nuclei and prominent nucleoli which is in agreement with other researches (5, 18). Although most researches focused on hepatocarcinogenesis of diethylnitrosamine (18, 19), there were no signs of carcinogenesis in liver sections and this difference may be due to dose and term of study. The activation of hepatic Stellate cells (HSCs) has been implicated in the pathogenesis of liver fibrosis (20). In the current study liver fibrosis is depicted by Masson’s trichrome.

This staining is an excellent technique to demonstrate the accumulation of collagen fibers in the liver tissue during hepatic fibrosis and cirrhosis (21). Liver sections of DEN + EPE group showed improved hepatocellular architecture with signs of recovery and decreased HSCs proliferation and collagen fibers. However moderate cell swelling was obvious. There are a lot of studies on immunomedultory, anti-inflammatory and anti- bacterial effects of *Echinacea purpurea* but scanty literature was available on protective effects of *Echinacea Purpurea* extract on hepatic and renal toxicity. Bayramoglu et al. investigated the effect of Echinacea on kidney and liver after experimental renal Ischemia / reperfusion injury in the rats. They showed that *Echinacea Purpurea* can decrease the concentrations of different liver enzymes and histopathologic changes such as inflammatory cell infiltration, necrosis, damage in hepatic cords and loss of intercellular border in liver (22) which are in agreement with the results of the current research. Ali (2008) reported that Echinacea extract has protective effects on the liver against cyproterone acetate. He mentioned antioxidant properties of *Echinacea purpurea* induced these effects (23). Ezz investigated the ameliorative effects of *Echinacea Purpurea* against gamma radiation induced oxidative stress and immune responses in male rats and reported that supplementation of *Echinacean purpurea* to rats during a period of 4 weeks did not affect the XOR system, as well as, the oxidant / antioxidant status of the spleen (24). Although in this research oxidative stress markers such as Superoxide dismutase were not assessed but the protective effects which were shown in this research may be related to antioxidant activity of this extract. Some studies have demonstrated that *Echinacea purpurea* roots are a good source of natural antioxidant and have free radical scavenging properties that could be used to prevent free- radicals effects (23, 25). The antioxidant activity of *Echinacea purpurea* could be ascribed to the polyphenolic components such as flavonoids, phenolic acids or phenolic diterpenes (25, 26) which shows the necessity to conduct more comprehensive studies on antioxidants effects of this extract on hepatic fibrosis. In summary, the present study suggested that *Echinacea Purpurea* extract exhibits relatively hepatoprotective and antifibrotic effects against DEN induced hepatotoxicity in rats.
